# Norms, rules and policy tools: understanding Article 5.3 as an instrument of tobacco control governance

**DOI:** 10.1136/tobaccocontrol-2021-057159

**Published:** 2022-04-07

**Authors:** Rob Ralston, Selamawit Hirpa, Shalini Bassi, Denis Male, Praveen Kumar, Rachel Ann Barry, Jeff Collin

**Affiliations:** 1 Global Health Policy Unit, Social Policy, School of Social and Political Science, University of Edinburgh, Edinburgh, UK; 2 SPECTRUM Consortium (Shaping Public Health Policies to Reduce Inequalities and Harm), London, UK; 3 Department of Preventive Medicine, School of Public Health, Addis Ababa University, Addis Ababa, Ethiopia; 4 HRIDAY, Delhi, India; 5 School of Public Health, Makerere University, Kampala, Uganda; 6 Department of Commerce, Manipal Academy of Higher Education, Manipal, Karnataka, India

**Keywords:** tobacco industry, global health, public policy

## Abstract

**Introduction:**

Article 5.3 of the WHO Framework Convention on Tobacco Control, elaborated via its implementation guidelines, can be understood as a policy instrument comprising norms, rules and policy tools designed to shape practices of policy making and minimise tobacco industry interference.

**Methods:**

This qualitative research is based on in-depth interviews with officials from diverse government sectors and non-governmental organisations across countries (Ethiopia, India, Uganda) that have adopted measures to implement Article 5.3.

**Results:**

The data highlight varied perceptions and knowledge of Article 5.3 norms between health and non-health sectors. Health officials typically link its *core* norm of a fundamental conflict between public health and industry interests to the *governance* norm of protecting public health policies from industry interference. While officials in sectors beyond health broadly endorsed this core norm, they exhibited more limited awareness of Article 5.3 and its model of governance. The results examine how rules to implement Article 5.3 have been codified, but identify the absence of policy tools necessary to operationalise rules and norms. This limitation, alongside restricted awareness beyond health departments, suggests that political commitments to implement Article 5.3 will have limited impact on practices of stakeholder consultation and policy engagement with the tobacco industry.

**Conclusion:**

Conceptualising Article 5.3 as a policy instrument helps to explain how its rules and policy tools interact with each other and with broader governance processes. This framework has the potential to enhance understanding of Article 5.3 and help identify opportunities and constraints in its implementation.

## Introduction

In setting and implementing their public health policies with respect to tobacco control, Parties shall act to protect these policies from commercial and other vested interests of the tobacco industry in accordance with national law.^1^

Article 5.3 of the WHO Framework Convention on Tobacco Control (FCTC) articulates the basis for a model of health governance that is distinctive within global health.^2^ In requiring participating countries to protect public health policies from tobacco industry interference, it explicitly addresses corporate attempts to undermine treaty objectives.^1^ Elaborating a series of recommendations to advance this objective, Article 5.3 implementation guidelines were unanimously adopted at the third WHO FCTC Conference of the Parties (CoP) in 2008.^3^ These represented a significant achievement amid highly politicised negotiations^4^ and a critical step towards developing an approach to tobacco control governance capable of supporting effective FCTC implementation.^2^

While Article 5.3 is viewed as the foundation upon which achievement of the FCTC’s wider goals depend,^5^ it has also remained at the margins of tobacco control debates.^6 7^ Existing research on Article 5.3 implementation highlights fragmented and inconsistent compliance with its provisions,^6–13^ yet there has been limited analysis of Article 5.3 from a governance perspective. The challenges of Article 5.3 have been primarily articulated from an advocacy lens of monitoring tobacco industry interference.^14 15^ Less attention has been paid to understanding Article 5.3 as a *policy instrument* (a set of rules and procedures governing the interactions and behaviours of actors and organisations^16^). This instrument asks policy makers to act in distinctive and potentially challenging ways that deviate from day-to-day practices of stakeholder engagement in other policy spheres. This suggests a need to examine how the different elements of Article 5.3 and its guidelines are configured and to explore the specific ways in which these have been operationalised.

The WHO guidelines for implementation of Article 5.3 set out 8 recommendations, with 34 subrecommendations, encompassing awareness raising; limiting industry interactions and ensuring transparency; rejecting partnerships and voluntary agreements; avoiding conflicts of interest; information provision; denormalising corporate social responsibility (CSR) activities; withholding preferential treatment; and treatment of state-owned interests.^3^ These incorporate different administrative and regulatory priorities for implementing Article 5.3 and, drawing on policy studies literature,^16–18^ collectively constitute a policy instrument comprising three key elements:

- *Norms* that establish key principles and a ‘logic of appropriateness’^19^ about the behaviour of government officials and their interactions with tobacco industry actors.
- *Rules* as mutually accepted or codified practices^20 21^ that guide policy actors by setting out which behaviours are required, permitted, restricted or precluded in developing public health policies.
- *Policy tools* as substantive and procedural instruments^16 17^ that frame and shape policy making by specifying mechanisms to operationalise norms and rules and advance policy goals.

**Figure 1:**
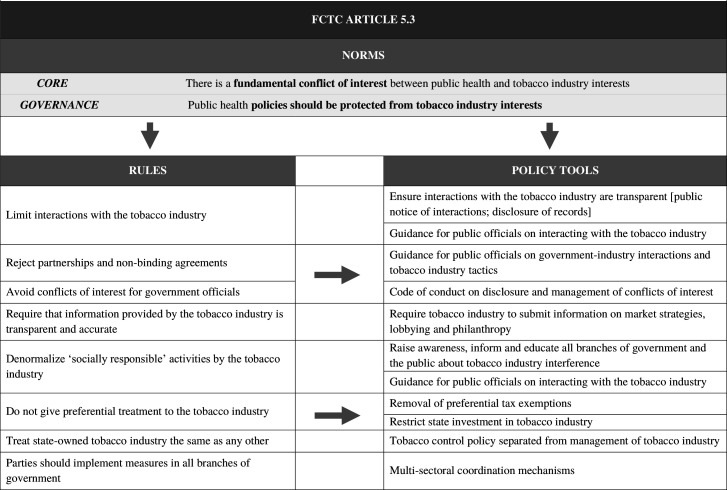
Norms, rules and policy tools of Article 5.3. FCTC, Framework Convention on Tobacco Control.

Focusing on the WHO guideline recommendations, we identify two *norms* that guide Article 5.3 as an instrument: a *core* norm identifying a fundamental conflict between the tobacco industry and public health interests, and a *governance* norm asserting that public health policy making should be protected from industry interference and vested interests. These norms are formalised in *rules*, elaborated in a series of recommended decision-making practices. These include requirements to minimise interactions, avoid conflicts of interest for government officials and avoid preferential treatment. The guidelines also envisage development of substantive *policy tools* to operationalise Article 5.3 and embed it in governance practices. Such tools^17^ aim to shape the ways in which policies are formulated and to manage any interactions that occur (eg, administrative processes to ensure transparency, guidelines that define the conduct of necessary interactions and codes of conduct for government officials). This framework is depicted in figure 1, mapping the implementation guidelines^3^ as a set of norms, rules and policy tools, informed by document analysis of the guidelines and of relevant policy documents in the contexts studied.^22–25^

The paper aims to explore the extent to which these elements of Article 5.3 have been applied in policy and practice via a comparative study of three countries (Ethiopia, India, Uganda) that have adopted measures to minimise industry interference. We first examine varied understandings and knowledge of Article 5.3 norms between health officials and other government sectors. It then assesses rules and procedures that have been adopted in attempting to redefine terms of engagement with the tobacco industry, before addressing the limited development of policy tools as mechanisms to support changes to the decision-making practices of policy makers. The discussion examines the implications of this framework for understanding the persistent challenges faced by Parties in effectively implementing Article 5.3 and reshaping established practices of stakeholder consultation and policy engagement with the tobacco industry.

## Methods

### Case selection

This research formed part of the Tobacco Control Capacity Programme (TCCP), an international consortium funded by the UK Global Challenges Research Fund to strengthen research capacity for tobacco control in low-and middle-income country (LMIC) contexts. It was guided by priorities identified by LMIC partner institutions and stakeholders, led by four project teams in Addis Ababa, Kampala, Delhi and Manipal, and Karnataka, and builds on case studies examining Article 5.3 implementation in these contexts.^26–29^ Preliminary findings were reviewed and key themes discussed at TCCP consortium meetings in London and Edinburgh, Addis Ababa, and New Delhi.

The case selection offers diverse policy trajectories and experiences through which to explore Article 5.3 implementation. Table 1 summarises the coverage of the WHO guidelines in policies adopted in Ethiopia, Uganda and India, with two research sites in Delhi and Manipal facilitating analysis of Article 5.3 implementation across multiple levels of government.

**Table 1:** Comparison of Article 5.3 codification across four low-and middle-income country (LMIC) contexts

		Uganda	Ethiopia	India	Karnataka state
Norms	Core				
	Governance				
Rules	Limit interactions				
	Reject partnership/non-binding agreements				
	Avoid conflicts of interest				
	Transparent industry information				
	Denormalise CSR				
	No preferential treatment				
	Treat state-owned industry the same				
	Whole-of-government applicability				
Policy tools	Transparency of interactions				
	Guidelines on necessary interaction				
	Code of conduct				
	Information disclosure by industry				
	Raise awareness				
	Removal of tax exemptions				
	Multisectoral coordination mechanisms				

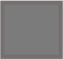
, broad consistency; 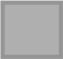, limited provision; 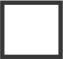, omitted.

CSR, corporate social responsibility.

Ethiopia’s 2019 tobacco control legislation codified several Article 5.3 guidelines in the context of the government’s negotiations with Japan Tobacco International over the privatisation of its state-owned tobacco monopoly.^26^ The adoption of Article 5.3 guideline recommendations in Uganda,^27^ an important leaf grower and exporter,^30^ is recognised as an example of good legislative practice.^31^ India’s federal system, in which central and state governments share constitutional responsibility for public health,^32 33^ has led to Article 5.3 ‘notifications’ being issued at the subnational level.^28^ Karnataka is one of 13 states (in 2019) to have adopted such measures, in which wider tensions between agriculture and health over local tobacco production are reflected in contrasting institutional interests and mandates.^29^ Building on subnational notifications, India’s Ministry of Health and Family Welfare (MoHFW) has adopted a National Code of Conduct for Public Officials in 2020 that applies to departments under its jurisdiction. Across these diverse political and institutional contexts, there is considerable overlap in what rules, procedures and policy tools have been included or omitted. The *governance* norm of protecting public health policies from tobacco industry interests is referenced in policy documents across all four contexts, and is accompanied by rules to limit interaction with the tobacco industry and avoid conflicts of interest for government officials. With the exception of Ethiopia, adopted policies reject partnerships and non-binding agreements, while only Uganda’s Tobacco Control Act requires that information provided by the tobacco industry is transparent, restricts preferential treatment and seeks to denormalise CSR initiatives.

The policies reviewed provide limited coverage of policy tools to embed norms and rules in governance practices. While tools to ensure transparency of government–industry interactions (such as public disclosure of meetings) have been adopted in all four contexts, only India has formalised guidelines for defining necessary interactions or developed a code of conduct to avoid conflicts of interest for government officials. Moreover, tools requiring disclosure of information on industry market strategies or corporate philanthropy are generally absent (although Uganda’s Act requires tobacco industry reporting to its National Coordinating Mechanism).

### Data collection and analysis

This paper is based on 115 semistructured interviews with government officials, stakeholders and health advocates in Ethiopia (n=21), Uganda (n=35) and across different levels of government in India (n=26), with a focus on the state of Karnataka (n=34). Summarised in table 2, the interviewees included policy makers from health departments and from other government sectors, including trade, finance and revenue, customs, development, environment, and agriculture. Interviewees varied in their background, seniority and policy roles, with positions spanning legal experts, mid-ranking and senior civil servants, policy specialists, economists, and enforcement officers. Further interviews were conducted with tobacco control advocates in non-governmental organisations (NGOs), officials in international organisations and academic researchers engaged in tobacco control policy debates. This approach ensured that diverse perspectives and experiences were captured in the data, with interviewees varying in experiences of policy making and of engagement with the industry. The semistructured interview guide was organised around three core themes: awareness of Article 5.3 and its norms; how rules and procedures had been operationalised; and perceptions about government–industry interactions. The semistructured approach allowed the interview schedule to be adapted across political and institutional contexts, with interviewees also asked context-specific questions.

**Table 2:** Summary of interviewees

Interviewee’s policy role	Sector	Interviewees (n)			
		Uganda	Ethiopia	India (code of conduct)	Karnataka (state notification)
National and/or local policy making	Health	4	4	10	14
	Environment	2			
	Finance and revenue	2	1	2	3
	Trade	3	1		
	Development	2			
	Customs		3		
	Food and Drug Administration		6		
	Education	2			5
	Agriculture	2			3
	Elected official	1	1		
	Executive		1	1	
	Other government agencies	6		2	5
Health advocates	NGOs	10	1	9	1
	Academic researcher		1		3
	International organisation	1	2	2	
		35	21	26	34

NGOs, non-governmental organisation.

SH, SB, DM and PK identified and selected interviewees based on publicly available information and indepth contextual knowledge of tobacco control. Interviewee selection was based in part on ‘snowball’ sampling,^34^ using professional networks and recommendations made by other interviewees. Interviews varied in length between 15 and 95 min (with most between 25 and 40 min), with interviews conducted in a private space inperson or via password-protected teleconferencing software. Interviewees were asked to review and sign a consent form allowing the interviews to be digitally recorded and for data to be used in research publications.

Fieldwork in Ethiopia and Uganda was completed between July and September 2019, prior to the COVID-19 pandemic. Research in India was significantly impacted by the pandemic and mitigation measures, notably travel restrictions and limited availability of government officials with additional COVID-19-related responsibilities. Interviews were conducted in English, Amharic (Ethiopia) and Kannada (a regional language in Karnataka) and the recordings translated, transcribed and anonymised. They were then coded in NVivo V.12 using a thematic framework developed via descriptive analysis, followed by conceptual coding of the interview data. Interviews were coordinated by SH, SB, DM and and transcripts coded by SH, SB, DM, RR, and RAB with input from JC. The research obtained ethical approval from the four in-country research institutions and the University of Edinburgh.

The analysis is informed by document analysis of the WHO guidelines for implementation of Article 5.3^3^ and of locally relevant policy documents.^22–25^ We draw on the policy studies literature on policy instruments^16 17^ in categorising the WHO guidelines’ 8 recommendations and 34 subrecommendations in terms of norms, rules and policy tools. Figure 1 provides a visual depiction of Article 5.3 organised according to these dimensions. This framework was used to map the extent of Article 5.3 implementation across the three case studies, assessing the presence of codified or formalised commitments in tobacco control legislation and administrative protocols.

## Results

### Contrasting perceptions of core and governance norms

The interview data suggest that policy officials in health departments were generally strongly supportive of both the core norm (of a fundamental conflict between public health and industry interests) and the governance norm (that public health policies should be protected from industry interference). Furthermore, these two norms were seen as connected and reinforcing, as a policy maker from the Ethiopian Food and Drug Agency argued:

As we have said previously there is irreconcilable interest. The public health policy interest is to decrease tobacco users to zero while the industry interest is maximizing the business. So, we can say that there is irreconcilable interest.

For policy makers in health departments, the existence of fundamental tensions between public health and tobacco industry interests required principles and procedures to manage government–industry interactions. As an official in Uganda’s Ministry of Health emphasised, “Article 5.3 is actually about protecting policies or shielding public health policies against any interference by the tobacco industry […] or those who front their interests.” In addition, many interviewees working in health departments and in health advocacy regarded Article 5.3 norms as underpinning the FCTC. In India, one civil society official reflected that:

The basic principle of [the] FCTC says there is an irreconcilable conflict between public health goals and the goals of the tobacco industry, then the motive is to protect public health policies from interference by the tobacco industry.

Such sentiments were echoed by others, including a legal expert in Uganda who felt that “Article 5.3 is the pivot of the whole FCTC” and that “you can barely implement any other Article of the FCTC if you don’t uphold Article 5.3, because the industry will always interfere.”

Interviewees working in health (and particularly in tobacco control) generally interpreted the governance norm of Article 5.3 as a response to fundamental conflicts. By contrast, interviewees from government sectors beyond health were consistently less likely to be aware of this norm or to perceive it as necessary for their work. Despite this, officials from sectors such as trade and environment did recognise tensions between public health and the tobacco industry’s core business model. As a senior civil servant in Uganda put it, “of course there is a conflict” as the “tobacco industry is securing a business and looking at profits.” Yet such perceptions were rarely (if at all) connected to the idea of minimising industry interference in policy making and subordinate to the primary objectives of other ministries. As one trade official in Uganda put it: “[a]s a ministry, of course, there is a conflict of interest, but you have to support the economy.”

### Rules to minimise tobacco industry interference

In seeking to protect against tobacco industry interference, governments in Ethiopia, Uganda and India have adopted rules that require agencies actively limit their interactions with the tobacco industry, permitting only those deemed necessary for policy making. The importance of such rules was stressed by several government officials in health departments and by advocates, who discussed Article 5.3 implementation in terms of insulating decision-making from the influence of tobacco industry actors. For example, a policy maker from Uganda’s health department reflected:

So, we don’t allow them in our policy formulation process. They are not involved in our law [or policy] formulation, they are not involved in the policy [or] even regulations. We try as much as possible to avoid them.

This rule was seen as linked to other implementation guidelines, such as rules to ensure transparency of any interactions with the tobacco industry. In Ethiopia one policy maker described implementing Article 5.3 as being centred around “procedural issues, like making it transparent and being accountable. For example, documenting the nature of the meeting, arranging the meeting, making [its] proceedings publicly accessible.” Similarly, a department of health official in Uganda noted that “if there is any engagement it has to be transparent.”

The interview data indicate that health officials viewed rules addressing conflicts of interest as crucial to implementing Article 5.3. Both Ethiopia and Uganda have adopted tobacco-specific rules to avoid conflict of interest for government officials, while this dimension was considered particularly important by officials in Karnataka and other Indian state governments. As a Karnataka-based interviewee remarked, “I mean, it’s logical that there should not be any conflict of interest […] it lays down the norms for reaching out or meeting the tobacco industry. So, it’s all about what norms and protocols are to be followed.”

Yet, while codified in tobacco control laws of Ethiopia and Uganda, and at different levels of government in India, the data suggest scepticism among health department officials about awareness in other government sectors. For example, one Ethiopia policy official noted that “most institutions may not know that they should not work [with the tobacco industry]. I can’t say that everyone knows about the FCTC – it’s only those working in [tobacco control] that have this knowledge.” This was echoed by a tobacco control consultant in Karnataka, who claimed that policy officials beyond health were “surprised that something like this exists” and that “only the people who are much [more] closely engaged, like health departments, are aware.” This view corresponds with interview data from other sectors, where most government officials reported limited knowledge of codified rules to implement Article 5.3. As interviewees from Karnataka and Uganda described:

This came to us from the department of health as a notification where we were told not to interact with the tobacco industry. But it came quite a [long] time ago and I don’t remember the details in it.

I don’t know that article – there are so many articles we deal with.

This uncertainty about Article 5.3 procedures contrasted with support for engagement and collaboration with the tobacco industry among interviewees in departments such as trade, revenue and customs, and finance. For example, a policy official within the Ethiopian Revenue and Customs Authority argued that:

There is a mutual interest in preventing illegal trade […] it is about working together and what you have to collaborate on. The government has to be in place to cooperate with the private sector, including tobacco.

Interactions don’t lead to interference. That’s my personal opinion […] Talking to someone is not interference. If I [work] in an administrative body and somebody comes to complain, then that [actor] has the right to express that and I have to listen to them as a public servant.

This preference for policy engagement with the tobacco industry was evident among some interviewees across all three country contexts, with a trade official in Uganda similarly claiming that the “tobacco industry is a stakeholder and a major player in the country’s economy. We need to engage them.” Indeed, while some interviewees acknowledged the risk of interference, they did not feel this precluded engagement and partnership with industry actors. As interviewees in Ethiopia and Uganda asserted:

The government has to be in a place to cooperate with the private sector, including tobacco […] but there should not be interference.

In the policy making process, there is provision for wide consultation […] So, whoever is affected by a law or policy you are bringing in must be listened to and a consensus must be taken into consideration […]. I wouldn’t allow interference; I would like to steer contribution in a consultative process.

### The neglected aspect of policy tools

This framework’s third element of Article 5.3 measures covers the use of substantive instruments or mechanisms as tools to translate norms and rules into practice. Such tools are designed to make policy operational, for example, through providing detailed guidance for deciding whether and how to interact with the tobacco industry or specifying changes to policy frameworks such as removing preferential tax exemptions. Despite the importance of tools in operationalising Article 5.3, these have often been neglected in tobacco control legislation and protocols, a gap that is visible across all policy contexts examined here (table 1).

First, while tobacco control legislation in both Ethiopia and Uganda seeks to limit government–industry interactions, this has apparently not been formalised into specific guidance on the conduct of public officials. The Ugandan Tobacco Control Act^24^ requires that government officials ‘shall not interact with the tobacco industry except where it is strictly necessary’. However, as the following NGO official describes, the government had not yet developed tools to implement this rule:

There is a loophole there, and this could be covered with regulations that stipulate guidelines for interactions – when interactions are necessary [and] to ensure effective transparency […] Yet the law as it stands, it says ‘to be transparent and accountable.’ I don’t know if the Ministry of Health has ever got a call from any [other] ministry saying, ‘the tobacco industry wants to have a meeting with us, how should we go about it?’ […] So, definitely there are some transparency issues that we need to tackle.

The gap between adopted rules and associated tools also limited Article 5.3 implementation in Ethiopia. According to one official, “there is no detail about measures that should be taken to prevent tobacco industry interference. Just based on Article 5.3, it was said that we should not have communication with the tobacco industry.” This contrasts with Article 5.3 implementation in India, where procedural tools such as guidance on interacting with the tobacco industry and codes of conduct for public officials have been integrated into both state-level notifications and a national code of conduct for health officials. The adoption of protocols appears to have had some impact on how public officials approach interaction with the tobacco industry, however. One state health official detailed how guidance had helped them to communicate to the tobacco industry that “interaction does not imply partnership.”

## Discussion

Conceptualising Article 5.3 and its guidelines as a policy instrument with three broad elements helps to illuminate key achievements, gaps and institutional constraints in its implementation in Ethiopia, India and Uganda. This framework offers scope to enhance both analysis of measures adopted to minimise tobacco industry interference and understanding of government officials’ experiences across diverse ministries and contexts.

Differentiating between the core norm of a fundamental conflict of interest and governance norm of minimising industry interference sheds new light on the familiar problem of limited engagement in ministries and government agencies beyond health.^6 7 35^ This highlights broad recognition of Article 5.3’s core norm among officials working in different policy sectors, by contrasts with more limited understanding and acceptance of the governance norm and its associated rules. While the widespread acknowledgement of a fundamental conflict of interest between tobacco industry and public health interests is a necessary step towards effective tobacco control governance, the critical challenge is to operationalise governance norms via rules and policy tools. This poses questions about the adequacy of mechanisms that shape government interactions with the tobacco industry.

A focus on rules and procedures to define appropriate behaviour by officials and policy makers illustrates the selective implementation of Article 5.3 guidelines.^6^ Policies adopted by Ethiopia, Uganda, India’s MoHFW, Karnataka, and several other state governments all include requirements to limit interactions with the tobacco industry and avoid conflicts of interest by government officials. By contrast, none of these jurisdictions defines expectations around ensuring that information provided by the tobacco industry is transparent and accurate or covering treatment of state-owned interests in the tobacco industry. The latter appears as a particularly significant omission, given the recent privatisation of the tobacco monopoly in Ethiopia and complex state interests in tobacco in India.^26 36^ Only Uganda’s legislation clearly implies that Article 5.3 obligations apply across all government departments. This suggests that the restricted scope of engagement with minimising industry interference beyond health is, in part, a corollary of limited rule development.

The limits of efforts to embed Article 5.3 norms in the day-to-day governance practices of officials appear most marked with respect to policy tools. The absence of measures on raising awareness from each of the policies examined suggests limited institutional commitment to make Article 5.3 operational across government departments. All four jurisdictions provided for transparency in reporting interactions with the tobacco industry, which implies that this measure is aimed at the ‘low hanging fruit’ of limited information disclosure rather than transformative change to practices. By contrast, development of mechanisms to support officials in defining ‘necessary interaction’ with the tobacco industry is limited; such measures are absent from legislation in Uganda and Ethiopia, while India’s code of conduct reproduces text from the guidelines rather than offering detailed assistance on how to interpret or operationalise them.^23^

Interpreted from a policy instrument approach, reviewing Article 5.3 measures adopted and omitted helps in focusing attention to the governance functions of specific rules and policy tools and how they complement and support each other. This framework helps to explain the policy work performed (or neglected) by different dimensions of this instrument, and how rules and tools interact with each other and with the broader context of policy making. The latter can encompass synergies or tensions with other norms, such as broader requirements for stakeholder consultation^26 37^ and also with other instruments. Existing literature on Article 5.3 implementation has noted the reliance on ‘passive implementation’, or addressing tobacco industry interference via existing mechanisms such as codes of conduct for public servants.^6 7^ The value of such an approach has been questioned amid a preference for tobacco industry-specific measures,^14 31^ but this analysis raises questions about this preference. If the core problem of Article 5.3 is the promotion of whole-of-government engagement, then tobacco industry-specific measures may reinforce the extent to which implementation is siloed and responsibility restricted to the ministry of health,^27^ as evident in India’s code of conduct for public servants being restricted to health officials.

While Article 5.3 studies have identified informal ad hoc working norms as substituting formal mechanisms,^6 7^ we suggest that norms and codified procedures are constitutive of Article 5.3 and complementary dimensions of this instrument.^38^ Indeed, the significance of Article 5.3 norms in articulating a fundamental conflict of interest and a commitment to reducing industry interference in policy making helps to explain its status among policy communities seeking to regulate other commercial determinants of health. Notwithstanding the generally poor track record of implementation, equivalents to Article 5.3 are seen as essential to progress in alcohol, food systems and nutrition, fossil fuels, and gambling.^39–44^ Yet, in many respects, Article 5.3 is a rather less developed policy instrument than other approaches to managing engagement with the commercial sector. This includes the WHO’s approach to prevent and manage conflicts of interest in nutrition policy,^45 46^ which entails a six-step decision-making procedure and detailed guidance on monitoring and evaluation. Another notable example is the Pan American Health Organization developing a triage instrument and a roadmap to embed the process in government decision-making processes.^47^ The policy tools and support available to assist officials in nutrition policy arguably exceed those in Article 5.3. In the absence of a clear consensus on conflict of interest between public health and the food industry, however, the nutrition tool lacks the normative basis of Article 5.3 as an obligation in an international legal treaty.^48^

The differences in these two policy instruments point to the significance of the process of negotiating Article 5.3 guidelines and to ways forward in supporting their more effective implementation. The nutrition tool was developed via a process that has included publication of a draft, online public consultation, revision and testing its applicability in diverse policy contexts.^46 47^ Article 5.3 implementation guidelines were, by contrast, elaborated via a working group, developed in meetings hosted by the governments of Netherlands and Brazil, and revised in the time-pressured and polarised context of CoP3 negotiations.^4 49 50^ The guidelines constitute an important political achievement, via a process perhaps better suited to the articulation of norms than the detailed revision of procedural rules and substantive policy tools necessary to establish an effective system of tobacco control governance. Technical support provided by the WHO and civil society^15 31 51^ and innovative tools developed by states to address specific issues^52–55^ have partially addressed such limitations.

The research and framework presented here indicates the need for further qualitative research to explore barriers specific to the adoption of different policy tools, particularly given the dearth of measures to address preferential treatment, state ownership and CSR, as well as the widely neglected rule specifying transparency in tobacco industry information. The interview data highlight the challenges that Article 5.3 poses to the daily operating procedures of policy makers and officials. Given that Ethiopia, Uganda and India tend to be considered as comparatively high-performing with regard to implementation, this questions a tendency within tobacco control to assume that this ‘should be simple’^56^ and suggests that participatory research with policy makers and advocates would be invaluable in addressing the gaps in rules and policy tools identified.

**What this paper adds**

- This study conceptualises Article 5.3 as a policy instrument comprising three key dimensions: *norms*, *rules* and *policy tools*.
- This framework provides important insights into Article 5.3 implementation, helping to explain how its constituent elements interact with each other and broader institutional contexts.
- Analysis of Article 5.3 implementation in Ethiopia, Uganda and India highlights selective implementation, in which rules to protect public health policies from tobacco industry interference have often not been operationalised in governance practices through policy tools.
- The achievement of Article 5.3 in establishing norms around a fundamental conflict between public health and tobacco industry interests and the need to protect public health policies from industry interference helps to explain its status among those seeking to regulate other commercial determinants of health.

## Data Availability

No data are available.

## References

[1] WHO . Who framework convention on tobacco control, 2003. Available: https://apps.who.int/iris/bitstream/handle/10665/42811/9241591013.pdf?sequence=1

[2] Collin J . Tobacco control, global health policy and development: towards policy coherence in global governance. Tob Control 2012;21:274–80. 10.1136/tobaccocontrol-2011-050418 22345267 PMC4474152

[3] World Health Organization . Who framework convention on tobacco control guidelines for implementation of article 5.3, 2008. Available: http://www.who.int/fctc/guidelines/article_5_3.pdf

[4] Mulvey K . A life-saving precedent: protecting public health policy against big tobacco. Tob Control 2010;19:95–7. 10.1136/tc.2009.032755 20378584

[5] Puska P , Daube M , WHO FCTC Impact Assessment Expert Group . Impact assessment of the who framework convention on tobacco control: introduction, general findings and discussion. Tob Control 2019;28:s81–3. 10.1136/tobaccocontrol-2018-054429 30181384 PMC6589462

[6] Fooks GJ , Smith J , Lee K , et al . Controlling corporate influence in health policy making? an assessment of the implementation of article 5.3 of the world Health organization framework convention on tobacco control. Global Health 2017;13:12. 10.1186/s12992-017-0234-8 28274267 PMC5343400

[7] Hawkins B , Holden C . European Union implementation of article 5.3 of the framework convention on tobacco control. Global Health 2018;14:79. 10.1186/s12992-018-0386-1 30071862 PMC6090908

[8] Assunta M , Dorotheo EU . SEATCA tobacco industry interference index: a tool for measuring implementation of who framework convention on tobacco control article 5.3. Tob Control 2016;25:313–8. 10.1136/tobaccocontrol-2014-051934 25908597 PMC4853530

[9] Lie JLY , Willemsen MC , de Vries NK , et al . The devil is in the detail: tobacco industry political influence in the Dutch implementation of the 2001 EU tobacco products directive. Tob Control 2016;25:545–50. 10.1136/tobaccocontrol-2015-052302 26349910

[10] Bialous SA . Impact of implementation of the who FCTC on the tobacco industry's behaviour. Tob Control 2019;28:s94–6. 10.1136/tobaccocontrol-2018-054808 30659104 PMC6589456

[11] Labonté R , Lencucha R , Goma F , et al . Consequences of policy incoherence: how Zambia’s post-FCTC investment policy stimulated tobacco production. J Public Health Policy 2019;40:286–91. 10.1057/s41271-019-00171-8 31053789 PMC7063566

[12] Lencucha R , Drope J , Chavez JJ . Whole-of-government approaches to NCDS: the case of the Philippines Interagency Committee-Tobacco. Health Policy Plan 2015;30:844–52. 10.1093/heapol/czu085 25096748

[13] Labonté R , Lencucha R , Drope J , et al . The institutional context of tobacco production in Zambia. Global Health 2018;14:5. 10.1186/s12992-018-0328-y 29338793 PMC5771190

[14] Assunta M . Global tobacco industry interference index. Bangkok, Thailand: Global Center for Good Governance in Tobacco Control (GGTC), 2020. https://exposetobacco.org/wp-content/uploads/GlobalTIIIndex2020_Report.pdf

[15] International Union Against Tuberculosis and Lung Diseases . The Union toolkit for who FCTC article 5.3: guidance for governments on preventing tobacco industry interference, 2012. Available: https://theunion.org/technical-publications/the-union-tookit-for-who-fctc-article-53-guidance-for-governments-on-preventing-tobacco-industry-interference

[16] Lascoumes P , Le Gales P . Introduction: Understanding Public Policy through Its Instruments?From the Nature of Instruments to the Sociology of Public Policy Instrumentation. Governance 2007;20:1–21. 10.1111/j.1468-0491.2007.00342.x

[17] Bali AS , Howlett M , Lewis JM , et al . Procedural policy tools in theory and practice. Policy and Society 2021;40:295–311. 10.1080/14494035.2021.1965379

[18] Simons A , Schniedermann A . The neglected politics behind evidence-based policy: shedding light on instrument constituency dynamics. policy polit 2021;49:513–29. 10.1332/030557321X16225469993170

[19] March J , March OJ , Olsen JP . Rediscovering institutions: the organizational basis of politics. New York: Free Press 1989, 1989.

[20] Powell WW , DiMaggio PJ . The new Institutionalism in organizational analysis. 2nd ed. Chicago: University of Chicago Press, 1991.

[21] Blanco I , Lowndes V , Salazar Y . Understanding institutional dynamics in participatory governance: how rules, practices and narratives combine to produce stability or diverge to create conditions for change. Crit Policy Stud 2021;66:1–20. 10.1080/19460171.2021.1984265

[22] Federal Democratic Republic of Ethiopia . Food and medicine administration Proclamation No. 112/2019, 2019. Available: https://www.tobaccocontrollaws.org/files/live/Ethiopia/Ethiopia%20-%202019%20Proclamation%20-%20national.pdf

[23] Ministry of Health & Family Welfare . Code of conduct for public Officials in compliance to article 5.3 of who FCTC, 2020. Available: https://landing.ggtc.world/dmdocuments/Code%20of%20Conduct%20for%20Public%20Officials%202.cdr.pdf

[24] Government of Uganda . Tobacco control act, 2015. Available: https://www.tobaccocontrollaws.org/files/live/Uganda/Uganda%20-%20TCA%20-%20national.pdf

[25] National Institute of cancer prevention and research, International Union against tuberculosis and lung diseases. notifications on article 5.3 of who FCTC, 2020. Available: http://smokelesstobaccocontrolindia.com/orders-notifications/#1592559640120-7af65f67-5bae [Accessed 1 Sep 2020].

[26] Hirpa S , Ralston R , Deressa W . 'They have a right to participate as a stakeholder': article 5.3 implementation and government interactions with the tobacco industry in Ethiopia. Tob Control 2022;31:s5–11. 10.1136/tobaccocontrol-2021-056885 35101970 PMC9125371

[27] Male D , Ralston R , Nyamurungi K . ‘That is a Ministry of Health thing’: Article 5.3 implementation in Uganda and the challenge of whole-of-government accountability. Tob Control 2022;31:s12–17. 10.1136/tobaccocontrol-2021-057049 35078911 PMC9125367

[28] Bassi S , Ralston R , Arora M . Understanding the dynamics of notification and implementation of Article 5.3 across India’s states and union territories. Tob Control 2022;31:s18–25. 35140171 10.1136/tobaccocontrol-2021-057119 PMC9125360

[29] Kumar P , Barry RA , Kulkarni MM . Institutional tensions, corporate social responsibility and district-level governance of tobacco industry interference: analysing challenges in local implementation of article 5.3 measures in Karnataka, India. Tob Control 2022;31:s26–32. 10.1136/tobaccocontrol-2021-057113 35078910 PMC9125366

[30] Appau A , Drope J , Labonté R , et al . Disentangling regional trade agreements, trade flows and tobacco affordability in sub-Saharan Africa. Global Health 2017;13:81. 10.1186/s12992-017-0305-x 29137678 PMC5686832

[31] Assunta M . Good country practices in the implementation of who FCTC article 5.3 and its guidelines, 2018. Available: https://www.who.int/fctc/publications/fctc-article-5-3-best-practices.pdf?ua=1

[32] Sharma CK , Swenden W . Continuity and change in contemporary Indian federalism. India Review 2017;16:1–13. 10.1080/14736489.2017.1279921

[33] Mondal S , Van Belle S , Bhojani U , et al . Policy processes in Multisectoral tobacco control in India: the role of institutional architecture, political engagement and legal interventions. Int J Health Policy Manag 2021;0. 10.34172/ijhpm.2021.66. [Epub ahead of print: 14 Jul 2021]. PMC9808220 34380195

[34] Farquharson K . A different kind of Snowball: identifying key policymakers. Int J Soc Res Methodol 2005;8:345–53. 10.1080/1364557042000203116

[35] Lencucha R , Drope J , Bialous SA . Institutions and the implementation of tobacco control in Brazil. Cad Saúde Pública 2017;33:e00168315. 10.1590/0102-311x00168315 29069213

[36] Rao NV , Bhojani U , Shekar P , et al . Conflicts of interest in tobacco control in India: an exploratory study. Tob Control 2016;25:715–8. 10.1136/tobaccocontrol-2015-052503 26612763

[37] Smith KE , Gilmore AB , Fooks G , et al . Tobacco industry attempts to undermine Article 5.3 and the "good governance" trap. Tob Control 2009;18:509–11. 10.1136/tc.2009.032300 19955541

[38] Drope J , Lencucha R . Evolving norms at the intersection of health and trade. J Health Polit Policy Law 2014;39:591–631. 10.1215/03616878-2682621 24603086 PMC4073791

[39] Moodie R , Stuckler D , Monteiro C , et al . Profits and pandemics: prevention of harmful effects of tobacco, alcohol, and ultra-processed food and drink industries. Lancet 2013;381:670–9. 10.1016/S0140-6736(12)62089-3 23410611

[40] Collin J . Taking steps toward coherent global governance of alcohol: the challenge and opportunity of managing conflict of interest. J Stud Alcohol Drugs 2021;82:387–94. 10.15288/jsad.2021.82.387 34100707

[41] David JL , Thomas SL , Randle M , et al . A public health advocacy approach for preventing and reducing gambling related harm. Aust N Z J Public Health 2020;44:14–19. 10.1111/1753-6405.12949 31777133

[42] Collin J , Hill SE , Kandlik Eltanani M , Eltanani MK , et al . Can public health reconcile profits and pandemics? an analysis of attitudes to commercial sector engagement in health policy and research. PLoS One 2017;12:e0182612. 10.1371/journal.pone.0182612 28886049 PMC5590731

[43] Marteau TM , Chater N , Garnett EE . Changing behaviour for net zero 2050. BMJ 2021;375:n2293. 10.1136/bmj.n2293 34615652 PMC8493657

[44] Dorado D , Monsalve S , Naik A , et al . Towards building comprehensive legal frameworks for corporate accountability in food governance. Development 2021:236–44. 10.1057/s41301-021-00319-8 34703162 PMC8531904

[45] World Health Organization . Who discussion paper. Safeguarding against possible conflicts of interest in nutrition programmes: draft approach for the prevention and management of conflicts of interest in the policy development and implementation of nutrition programmes at country level, 2017. Available: https://www.who.int/nutrition/consultation-doi/Discussion-paper-nutrition.pdf

[46] Ralston R , Hil SE , da Silva Gomes F , et al . Towards preventing and managing conflict of interest in nutrition policy? an analysis of submissions to a consultation on a draft who tool. Int J Health Policy Manag 2020. 10.34172/ijhpm.2020.52 PMC9056191 32610752

[47] Pan American Health Organization . Preventing and Managing Conflicts of Interests in Country-level Nutrition Programs: A Roadmap for Implementing the World Health Organization’s Draft Approach in the Americas, 2021. Available: https://iris.paho.org/bitstream/handle/10665.2/55055/PAHONMHRF210014_eng.pdf?sequence=1&isAllowed=y

[48] McHardy J . The who FCTC's lessons for addressing the commercial determinants of health. Health Promot Int 2021;36:i39–52. 10.1093/heapro/daab143 34897446 PMC8667548

[49] World Health Organization . Elaboration of guidelines for implementation of article 5.3 of the convention, 2008. Available: https://apps.who.int/gb/fctc/PDF/cop3/FCTC_COP3_5-en.pdf

[50] Framework Convention Alliance . Briefing 2: adoption of guidelines for implementation of article 5.3 (protection of public health policies with respect to tobacco control from commercial and other vested interests of the tobacco industry), 2008. Available: https://www.fctc.org/wp-content/uploads/2018/05/COP3_Article_5_3_-briefing.pdf

[51] World Health Organization . Technical resource for country implementation of who framework convention on tobacco control article 5.3 on the protection of public health policies with respect to tobacco control from commercial and other vested interests of the tobacco industry. Geneva, Switzerland: World Health Organization, 2012. http://apps.who.int/iris/bitstream/10665/44880/1/9789241503730_eng.pdf

[52] Australian Government Department of Health . Guidance for public officials on interacting with the tobacco industry, 2019. Available: https://www.health.gov.au/resources/publications/guidance-for-public-officials-on-interacting-with-the-tobacco-industry

[53] Civil Service Commission,, Department of Health . Joint memorandum circular No. 2010-01: protection of the bureaucracy against tobacco industry interference, 2010. Available: https://www.tobaccocontrollaws.org/files/live/Philippines/Philippines%20-%20JMC%202010-01%20-%20national.pdf

[54] Department of Health, . Foreign & Commonwealth Office. United Kingdom’s revised guidelines for overseas posts on support to the tobacco industry, 2013. Available: https://www.tobaccocontrollaws.org/files/live/England/England%20-%20UK%205.3%20Guidelines%20-%20national.pdf

[55] Department of Disease Control . Gazette: how to contact tobacco entrepreneurs and related persons B.E. 2553, 2010. Available: https://www.tobaccocontrollaws.org/files/live/Thailand/Thailand%20-%20Dept.%20Dis.%20Ctrl.%20on%20Contact%20with%20TI.pdf

[56] Gilmore AB , Fooks G , Drope J , et al . Exposing and addressing tobacco industry conduct in low-income and middle-income countries. Lancet 2015;385:1029–43. 10.1016/S0140-6736(15)60312-9 25784350 PMC4382920

